# MultiDCoX: Multi-factor analysis of differential co-expression

**DOI:** 10.1186/s12859-017-1963-7

**Published:** 2017-12-28

**Authors:** Herty Liany, Jagath C. Rajapakse, R. Krishna Murthy Karuturi

**Affiliations:** 1 School of Computing, National University of Singapore, 21 Lower Kent Ridge Rd, Singapore, 119077 Singapore; 2 Computational and System Biology, Genome Institute of Singapore, A-STAR, 60 Biopolis Street, Singapore, 138672 Singapore; 3 The Jackson Laboratory, 10 Discovery Dr, Farmington, CT 06032 USA; 4 School of Computer Science and Engineering, Nanyang Technological University, 50 Nanyang Ave, Singapore, 639798 Singapore

**Keywords:** Differential co-expression, Gene expression, MultiDCoX, Multi-factor analysis

## Abstract

**Background:**

Differential co-expression (DCX) signifies change in degree of co-expression of a set of genes among different biological conditions. It has been used to identify differential co-expression networks or interactomes. Many algorithms have been developed for single-factor differential co-expression analysis and applied in a variety of studies. However, in many studies, the samples are characterized by multiple factors such as genetic markers, clinical variables and treatments. No algorithm or methodology is available for multi-factor analysis of differential co-expression.

**Results:**

We developed a novel formulation and a computationally efficient greedy search algorithm called MultiDCoX to perform multi-factor differential co-expression analysis. Simulated data analysis demonstrates that the algorithm can effectively elicit differentially co-expressed (DCX) gene sets and quantify the influence of each factor on co-expression. MultiDCoX analysis of a breast cancer dataset identified interesting biologically meaningful differentially co-expressed (DCX) gene sets along with genetic and clinical factors that influenced the respective differential co-expression.

**Conclusions:**

MultiDCoX is a space and time efficient procedure to identify differentially co-expressed gene sets and successfully identify influence of individual factors on differential co-expression.

**Electronic supplementary material:**

The online version of this article (10.1186/s12859-017-1963-7) contains supplementary material, which is available to authorized users.

## Background

**Fig. 1:**
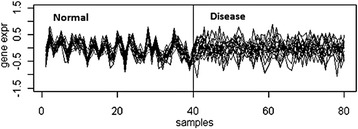
Differential Co-Expression. Geneset is co-expressed in normal samples but not in disease samples

Differential co-expression of a set of genes is the change in their degree of co-expression among two or more relevant biological conditions [1], illustrated in Fig. 1 for two conditions. Differential co-expression signifies loss of control of factor(s) over the respective downstream genes in a set of samples compared to the samples in which the gene set is co-expressed or variable influence of a factor in one set of samples over the other. This could also be due to a latent factor which had a significant influence on gene expression in a particular condition [2].

Since the proposal by Kostka & Spang [1], many algorithms have been developed to identify *differentially co-expressed* (referred as DCX throughout the paper) gene sets and quantify differential co-expression. The algorithms can be classified based on two criteria: (1) method of identification of DCX gene sets (targeted, semi-targeted and untargeted); and (2) scoring method of differential co-expression (gene set scoring and gene-pair scoring).

Based on the method of identification, similar to the one described by Tesson et al. [3], the algorithms can be classified into *targeted*, *semi-targeted* and *untargeted* algorithms. The *Targeted algorithms* [4] perform differential co-expression analysis on predefined sets of genes. The candidate gene sets may be obtained from public databases such as GO categories and KEGG pathways. They do not find novel DCX gene sets. Another disadvantage of targeted methods is their reduced sensitivity if only a subset of the given gene set is differentially co-expressed which results in the DCX signal diluted. In addition, the DCX gene sets that are composed of genes of multiple biological processes or functions may not be identified at all [2]. The *semi-targeted algorithms* [5, 6] work on the observation that the DCX genes are co-expressed in one group of samples. Hence, they perform clustering of genes in one set of samples, identify gene sets tightly co-expressed and test for their differential co-expression using the remaining group of samples. Although semi-targeted algorithms can identify novel gene sets, their applicability is limited to the co-expressed sets identified by the clustering algorithm. In addition, this approach also may suffer from lower sensitivity due to diluted DCX signal, similar to in targeted approach. On the other hand, the *untargeted algorithms* [1, 3, 7, 8] assume no prior candidate sets of genes and instead find the gene sets de novo and therefore have a high potential to identify novel gene sets without diluting DCX signal. The major drawback of untargeted approach is higher false discovery rate and computational requirements.

The second aspect of DCX gene set identification algorithms is the methodology employed in scoring differential co-expression of a given gene set: (1) gene set scoring or set-wise method, and (2) gene pair scoring. In *gene set scoring*, all genes are considered in the scoring at once such as in the linear modelling used in Kostka & Spang [1] and Prieto et al. [7]. On the other hand, *gene-pair scoring*, as used in DiffFNs [8] and DiffCoEx [3], computes differential correlation of each pair of genes in the gene set and summarizes them to obtain DCX score for the gene set. Gene pair scoring is intuitive and amenable to network like visualization and interpretation in single factor analysis settings. The first few methods (e.g. Kostka & Spang [1] and Prieto et al. [7]) are untargeted set-wise methods, while DiffFNs [8] is an untargeted gene-pair scoring method. However, many later methods, including an early method (DCA [5]) are predominantly targeted or semi-targeted algorithms using gene pair scoring. Differential co-expression has been used in various disease studies and identified many interesting changed interactomes of genes among different disease conditions. DiffFNs [8], Differential co-expression analysis [9], TSPG [10], and Topology-based cancer classification [11] were applied for the classification of tumor samples using interactome features identified using differential co-expression and shown good results over using individual gene features. The application of Ray and Zhang’s co-expression network using PCC and topological overlap on Alzheimer’s data helped identify gene sets whose co-expression changes in Alzheimer’s patients [12]. The multi-group time-course study on ageing [13] has identified gene sets whose co-expression is modulated by ageing. Application on data of *Shewanella oneidens* identified a network of transcriptional regulatory relationships between chemotaxis and electron transfer pathways [14]. Many other studies have also shown the significant utility of application of differential co-expression analysis [15–18]. However, none of the existing algorithms allow direct multi-factor analysis of differential co-expression, i.e. deconvolving and quantifying the influence of different biological, environmental and clinical factors of relevance on the change in co-expression of gene sets. Multi-factor differential co-expression analysis is important in many practical settings since each sample is characterized by many factors (a.k.a. co-factors) such as environmental variables, genetic markers, genotypes, phenotypes and treatments. For example, a lung cancer sample may be characterized by *EGFR* expression [19], smoking status of the patient, *KRAS* mutation and age. Similarly, ageing of skin may depend on age, exposure to sun, race and sex [20]. Deconvolving and quantifying the effects of these factors on gene set’s co-expression and eliciting relevant regulatory pathways is an important task towards understanding the change in the cellular state and the underlying biology of interest. In such a case, single-factor differential co-expression analysis suffers from multitude of tests and the interpretation of the gene sets may be cumbersome and misleading. Hence, we propose a very first methodology for such purpose called Multi-Factor Analysis of Differential Co-eXpression or MultiDCoX, a *gene set scoring* based *untargeted* algorithm. MultiDCoX performs greedy search for gene sets that maximize absolute coefficients of co-factors (as suggested in our earlier work [21]) in a linear model, while minimizing residuals for each geneset. The analysis of several simulated datasets demonstrate that the algorithm can be used to reliably identify DCX gene sets, and deconvolve and quantify the influence of multiple cofactors on the co-expression of a DCX geneset in the background of large set of non-DCX gene sets. The algorithm performed well even for genesets with weak signal-to-noise ratio. The analysis of a breast cancer gene expression dataset revealed interesting biologically meaningful DCX gene sets and their relationship with the relevant co-factors. Furthermore, we have shown that the co-expression of CXCL13 is not only due to the Grade of the tumor as identified in [22], but also could be influenced by ER status. Similarly, MMP1 appears to play role in two different contexts defined by more than one co-factor. These together demonstrate the importance of multi-factor analysis.

## Methods

### MultiDCoX formulation and algorithm

MultiDCoX procedure consists of two major steps: (1) identifying DCX gene sets and obtaining respective DCX profiles; and (2) identifying covariates that influence differential co-expression of each DCX gene set. The formulation essential to carry out these two steps is as follows.

Let *E*_*im*_ denote expression of gene *g*_*i*_ in sample *S*_*m*_. The co-factor vector characterizing *S*_*m*_ is denoted by *B*_*m*_ *= (B*_*m1*_ *, B*_*m2*_ *, B*_*m3*_ *,…,B*_*mz*_ *)* where *B*_*mk*_ is the value of *k* ^th^ factor for *S*_*m*_ which is either a binary or an ordinal variable. A categorical co-factor can be converted into as many binary variables as one less the number of categories of the factor. A real valued cofactor can be discretized into reasonably number of levels and be treated as ordinal variable.

We define a new variable *A*_*mn*_ *(I)* to summarize co-expression of gene set *I* between sample pair *S*_*m*_ and *S*_*n*_ for which *B*_*m*_ *= B*_*n*_ as

1 $$ {\displaystyle \begin{array}{l}{A}_{mn}(I)={\left(\frac{1}{\left|I\right|}{\sum}_{i=1}^{\left|I\right|}\left({E}_{im}-{E}_{in}\right)\right)}^2\\ {}{B}_{mn}={B}_m={B}_n\end{array}} $$

**Fig. 2:**
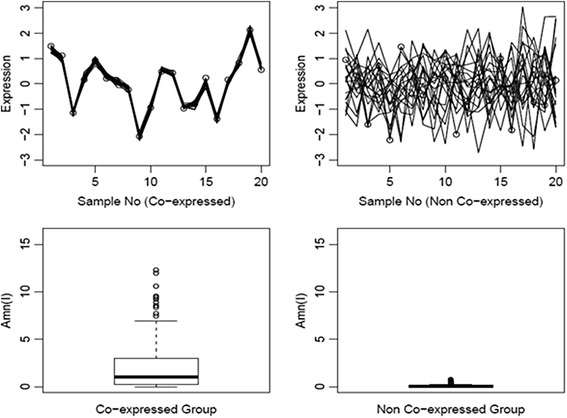
Illustration of A_mn_(I) for co-expression and non co-expression. *A*_*mn*_ *(I)* tends to be higher for tighter co-expression of a geneset, while it is close to 0 for no co-expression as illustrated by the boxplots for presence and absence of co-expression of genesets

*A*_*mn*_ *(I)* measures square of mean change of expression of all genes in *I* from *S*_*m*_ to *S*_*n,*_ i.e. measuring correlation between two samples over geneset *I.* Most of *A*_*mn*_ *(I)‘s* are expected to be non-zero among a group of samples in which *I* is co-expressed. On the other hand, if genes in *I* are not co-expressed in a group of samples then *A*_*mn*_ *(I)‘s* tend to be closer to zero as illustrated in Fig. 2.

We quantify the influence of the co-factors by fitting a linear model between *A*_*mn*_ *(I)s* and *B*_*mn*_s. In other words, *A*_*mn*_ *(I)s* are the instances of the response variable *A(I)*, *B*_*mn*_s form design matrix (B) and factors in the *B*_*mn*_s are explanatory variables or co-factors (F).

2 $$ A(I)\sim BF $$

The coefficient vector obtained from the above modelling (*Eq2*) is called differential co-expression profile of the gene set *I*, denoted by *F(I)*. *A(I), B* and *F* are of *a*x*1, a*x*z* and *z*x*1* dimensions respectively. Where ‘*a*’ is number of sample pairs which satisfy the condition in *Eq1* or subset of these sample pairs sampled for modelling, whichever is lower; *z* is number of factors in the model.

**Fig. 3:**
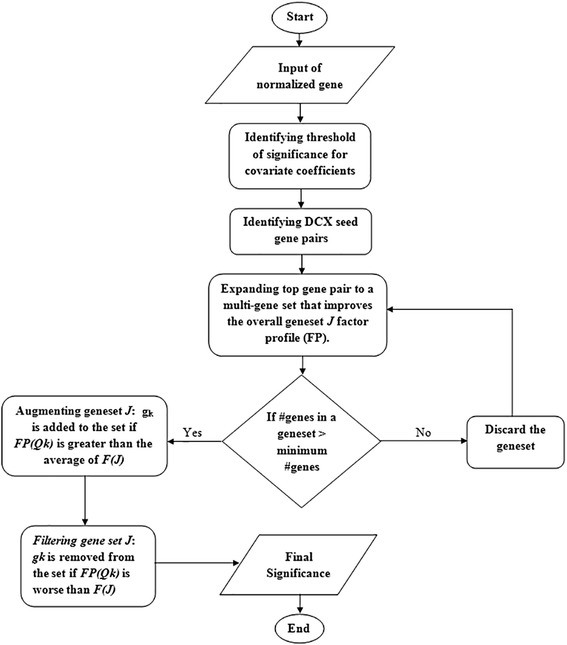
Flowchart of MultiDCoX algorithm. It captures all four steps of the algorithm, which are applied on a dataset until no additional DCX geneset is identified

The MultiDCoX algorithm identifies DCX gene sets by iteratively optimizing coefficient of a co-factor as outlined in Fig. 3: (1) setting significance threshold for co-factor coefficients; (2) choosing seed pairs of genes that demonstrate significant coefficient for the co-factor under consideration, i.e. the gene pairs may be differentially co-expressed for the co-factor; (3) expanding each chosen seed gene pair into a conservative multi-gene set by optimizing the respective coefficient; (4) augmenting the geneset to increase sensitivity or reduce false negatives while keeping the respective co-factor coefficient significant; and, (5) filtering out weak contributing genes from each geneset to increase specificity or reduce false positives. Each of these steps is explained in detail below.

1. *Setting threshold of significance for cofactor coefficients:* We generate the distribution of coefficients of the co-factors in *F* by random sampling of gene pairs: randomly sample large number of gene pairs, fit the linear model in *Eq2* for each pair and obtain the coefficients in the linear models. Pool absolute values of coefficients of all factors of all gene pairs, and set half of the *m* ^*t*h^ (*m* = 10 in our experiments) highest value as absolute threshold of significance for all co-factors. In other words,

$$ {C}_T={m}^{th}\ \mathit{\operatorname{Max}}\ {\bigcup}_l\ {\bigcup}_k\ \left\{|{F}_k\ \left({I}_l\right)\ |/2\right\} $$

where *F*_*k*_ *(I*_*l*_ *)* is coefficient on gene set (a pair of genes in this case) *I*_*l*_ for *k* ^*th*^ factor.

*T*_*oi*_ is the threshold for co-factor ‘*i*’ for geneset *I* and ‘*o*’ stands for ‘*original’*, derived from *C*_*T*_ as follows

$$ {T}_{oi}={C}_T\ \mathrm{if}\ {F}_i\ (I)>0=-{C}_T\ \mathrm{if}\ {F}_i\ (I)< 0 $$

The division by 2 is necessary to avoid damagingly strict threshold and lay wider net at the beginning of the algorithm. *m* > 1 is required as some of the sampled gene pairs could belong to DCX genesets which may overestimate the threshold and reduce sensitivity of the algorithm.

2. *Identifying DCX seed gene pairs:* For each gene, search is performed throughout the dataset to find its partner gene whose pair can result in a linear model (*Eq2*) with at least one significant cofactor. A cofactor is considered to be significant if its linear model F-test *p*-value is <0.01 and absolute value of its coefficient > *C*_*T*_. If no partner gene could be found, then the gene will be filtered out from the dataset to improve the computational speed at later stages of the algorithm. We have implemented this step using the procedure: (a) batch application of *qr.coef()* in *R-package* which computes only linear model coefficients using one QR decomposition, (b) filter out gene pairs whose linear model coefficients are in the range [*−C*_*T*_ *, C*_*T*_], (c) apply *lm()* on the gene pairs remaining after step ‘b’ to compute F-test *p*-values, and (d) further filter out gene pairs which do not meet requirements for the coefficient p-value. The batch application of *qr.coef()* is multi-fold faster than *lm()*. We use similar strategy in the steps 3.A-3.C below to reduce computational requirements compared to the direct application of *lm()*.

3. *Identifying DCX gene sets*: We optimize coefficient of each significant co-factor for each gene pair in the direction, in positive or negative direction, depending on the sign of the coefficient i.e. if the coefficient is negative (positive) its minimized (maximized). To do so, for each factor, the steps 3.A-3.C are iterated until all seed pairs for which the factor is significant are exhausted from the seed pairs obtained in the step 2.

3.A. *Expanding top gene pair to a multi-gene set*: We choose the gene pair whose constituent genes are not part of any of the multi-gene sets identified and whose linear model fit resulted in the highest coefficient for the co-factor of interest. It will be expanded to multi-gene set by adding genes that improve the coefficient of that co-factor in the direction of its coefficient for the gene pair. A sequential search is performed from first gene to the last gene in the data (the order of the genes will be randomized prior to this search). A gene is added to the set if it improved the coefficient of the co-factor under consideration i.e. the threshold to add a gene thereby the stringency increases as the search proceeds. The final set obtained at the end of this step is denoted by *J*. This step results in a most conservative DCX gene set. Factor profile *FP(J)* of *J* is defined as set of (*f*_*i*_ *,h*_*i*_) pairs as follows:

$$ FP\left(J,{T}_{oi}\right)=\left\{\left({f}_i, 1\right)\ |\ {F}_i(J)>{T}_{oi}\  AND\ P-{val}_i(J)< 0.01\right\}\bigcup \left\{\left({f}_i, 0\right)\ |\ |{F}_i(J)|\le |{T}_{oi}|\  OR\ P-{val}_i(J)\ge 0.01\right\}\bigcup \left\{\left({f}_i,- 1\right)\ |\ {F}_i(J)<-{T}_{oi}\  AND\ P-{val}_i(J)< 0.01\right\} $$

Where *f*_*i*_ is factor *‘i’* and *h*_*i*_ denotes whether it is positively (*h*_*i*_ *= 1*) or negatively (*h*_*i*_ *= −1*) significant or insignificant (*h*_*i*_ *= 0*):

*F*_*i*_ *(J)* is coefficient of factor *f*_*i*_ for gene set *J.*

*P-val*_*i*_ *(J)* is *p*-value of *F*_*i*_ *(J).*

3.B. *Augmenting gene set J*: As we tried to improve the coefficient of the co-factor for each addition of a gene in the expansion step (3.A), we may have missed many true positives which are not as strong constituents of *J*, but could be significant contributors. Therefore, we perform augmentation step to elicit some of the potential not-so strong constituents of *J* while preserving the factor profile of *J*. As the gene set identified in step (3.A) is conservative, we set a new threshold *T*_*ni*_ *(J)* or simply *T*_*ni*_ for the coefficient *F*_*i*_ *(J)* of each *f*_*i*_as

$$ {T}_{ni}(J)= Sign\left({F}_i(J)\ \right)\left(\alpha |{T}_{oi}|+\left( 1-\alpha \right)|\ {F}_i(J)\ |\right), 0\le \alpha \le 1\ \mathrm{if}\mid {h}_i\mid = 1;=\mid {T}_{oi}\mid, otherwise. $$

*T*_*ni*_ *(J)* will be as stringent as *T*_*oi*_ and at most equal to *F*_*i*_ *(J)* which is the coefficient obtained at the end of step (3.A). Moreover, we define centroid *E*_*C*_ *(J) = {E*_*Cm*_ *(J)}* of *J* as

$$ {E}_{cm}(J)=\frac{1}{\left|J\right|}{\sum}_{i\in J}{E}_{im} $$

*E*_*C*_ *(J) = [E*_*c1*_ *(J), E*_*c2*_ *(J),…,E*_*cs*_ *(J)]* is treated as a representative gene expression profile of *J* and find a gene sub set *K* such that each gene in *K, g*_*k*_ *,* the pair *K*_*k*_ *= (g*_*k*_ *, E*_*C*_ *(J))* satisfies the condition

$$ FP\left({K}_{k,}\ {T}_{ni}\right)= FP\left(J,{T}_{oi}\right)\ \mathrm{i}.\mathrm{e}.K=\left\{{g}_k\ |\  FP\left({K}_k,{T}_{ni}\right)= FP\left(J,{T}_{oi}\right)\right\} $$

Then the augmented set *L = J* ⋃ *K* as new DCX gene set.

**Fig. 4:**
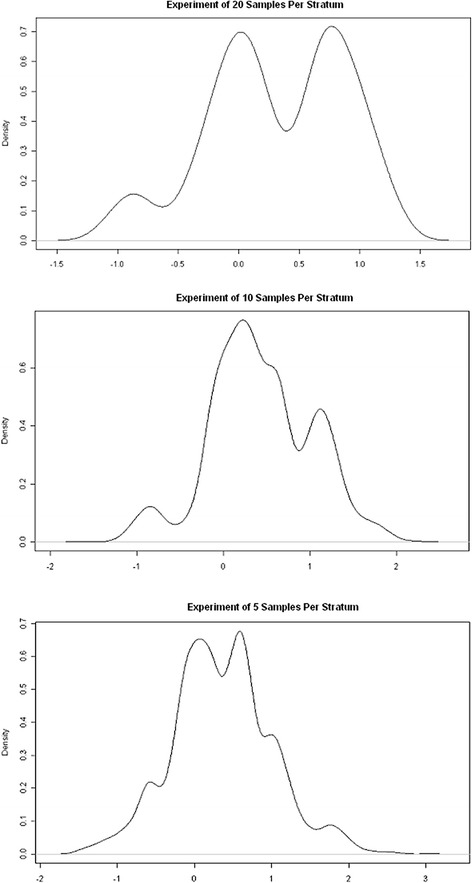
Illustration of selection of thresholds of significance for coefficients. Density plots of all coefficients (of the simulation data) resulted by MultiDCoX model fitting for varying number of sample/stratum. Thresholds are chosen to be first valleys either side of the central peak

3. C. *Filtering gene set L*: The set *L* obtained after the step (3.B) may contain false positives which can be filtered out as follows: As in the augmentation step, we compute *E*_*C*_ *(L*_*k*_ *), L*_*k*_ *= L-{k},* and evaluate each gene pair Q_k_ ∈ {(g_k_, *E*_*C*_ *(L*_*k*_ *)*) *| g*_*k*_ ∈ *L}* for *F(Q*_*k*_ *)*. *g*_*k*_ is removed from the set if |*F*_*i*_ *(Q*_*k*_ *)|* > |*F*_*i*_ *(L)|* for all *|hi| = 1*. Then the final gene set *R = {g*_*k*_ *| g*_*k*_ ∈ *L and |F(Q*_*k*_ *)|* < |*F(L)|}*. *R* is the final set output for the run.

4. *Identifying cofactors significantly influencing DCX of each gene set*: It is important to identify the factors influencing the DCX of a geneset (i.e. *FP(R))* to elicit underlying biology. The F-test *p*-value obtained for each cofactor by the linear model fit (in Eq2) in the above procedure need to be further examined owing to the dependencies among the gene sets explored. Therefore, we mark a co-factor to be influential (|hi| =1) on co-expression of *R* if it satisfies the following two criteria:

- *Effect size criterion*: We pool coefficients of all factors on all gene sets identified (denoted as *C*_*R*_) and examine their distribution. The valleys close to zero on either side of the central peak are chosen as the significance threshold *T*_*f+*_ and *T*_*f-*_, see Fig. 4 for illustration. The central peak is the result of the tests that signify chance association between the respective co-factor and co-expression of genesets. Whereas, the peaks on either side of the central peak signify coefficients of significant effects in testing/model-fitting. The valleys are identified by *T*_*f+*_ and *T*_*f-*_, which are good thresholds to call coefficients significant i.e. *F*_*i*_ *(R)* is considered to be significant if it is > *T*_*f+*_ or < *T*_*f-*_. The underlying assumption is that not all factors influence all gene sets and the coefficients of the co-factors with no or little influence on certain gene sets will be suggestive of the distribution of the coefficients under null hypothesis.
- *Permutation p-value criterion*: We permute the factor values of a DCX gene set (i.e. permute columns of B_mk_ matrix) and fit the linear model in *Eq2* for each gene set *R*. We repeat this procedure for a predefined number of iterations. A factor is said to be non-influential on the co-expression of the gene set under consideration if a minimum predefined fraction of permutations (0.01 in this paper) resulted in a fit in which the coefficient is better than *F*_*i*_ *(R)* and its F-test p-value is better than the F-test p-value of the coefficient without permutation or 0.01 whichever is lower.

Finally, the gene sets with at least one significant co-factor and of predefined size (i.e. at least 6 genes in the set) will be output as DCX gene sets along with their factor profiles.

#### Reducing computational and space requirements

Computational and space requirements can be further reduced using the following strategies: (1) Filter out genes with no detectable signals among almost all samples and genes that demonstrate very little variance across the samples. This can filter out up to 50% of the genes from the analysis. As a result, we can accomplish modest reduction in space requirement and substantial reduction in computational requirement as the search procedure is at least of quadratic complexity in time; (2) Further reduction in computational time can be achieved in the step 2 i.e. identifying seed gene pairs. Randomly split the genes into two halves and search for possible pairs where one belongs to one half and the other belongs to the other half, instead of all possible gene pairs. As many DCX genesets are expected to be sufficiently large, >10 genes, each split set is expected to contain >2 genes from each DCX geneset. This reduces computational time to find seed gene pairs by 2 fold. (3) Another possibility is to consider only a subset of sample pairs by randomly sampling a small fraction of (*m,n*)s for the linear model, it could be as small as 10% of all (*m,n*)s. These three strategies put together with the optimization described in the step 2 of MultiDCoX can massively reduce the space and computational requirement by several folds and make the algorithm practical.

## Results

### Simulation results

To evaluate efficacy of MultiDCoX, we analyzed simulated datasets of varying degrees of signal-to-noise ratio and sample size. Each simulated dataset consists of 50,000 probes as in a typical microarray and three factors of 12 stratums. Sample sizes were chosen to be either 60 or 120 or 240 i.e. 5, 10 and 20 samples per stratum respectively. Two factors *B1* and *B2* were binary (*∈ {−1, 1}*) and the other (B3) is an ordinal variable of three levels (*∈ {−1, 0, 1}*). Sample labels were randomly chosen for each factor and gene expression (*E*_*im*_) was simulated as described below:

$$ {E}_{im}={B1}_{im}+{B2}_{im}+{B3}_{im}+{O}_{im}+{e}_{im} $$

*B1*_*im*_ *= B1*_*m*_ ~ N(0,1) if *S*_*m*_ is in co-expressed group of *B1* and *g*_*i*_ is in DCX gene set for the factor *B1,* 0 otherwise. Similar interpretation holds for the remaining factors, B2 and B3, too. *O*_*im*_ *= O*_*m*_ ~ N(0,1) indicates co-expression over all samples if *g*_*i*_ belongs to set of genes co-expressed across all samples irrespective of the factor values. *E*_*im*_ *~ N(0,σ* ^*2*^ *)* is noise term and *σ* ^*2*^ is the extant of noise in the data.

We simulated 20 genes which show co-expression for *B1*_*m*_ *= 1* and *B2*_*m*_ *= 1*, 20 genes co-expressed for *B1*_*m*_ *= −1* only, and another 20 genes with *O*_*i*_ *= 1* only. With this we have two sets of negative controls: large number of genes with no co-expression and a set of 20 genes co-expressed across all samples. Ideally, a DCX geneset identification algorithm should be able to discriminate the first two sets of genes from the two control (negative) sets. Furthermore, we have tested our MultiDCoX for three different values of *σ ∈ {0.2, 0.5, 0.8}* i.e. from low noise to the noise comparable to the signal. We carried out 10 simulations for each choice of *σ*.

**Fig. 5:**
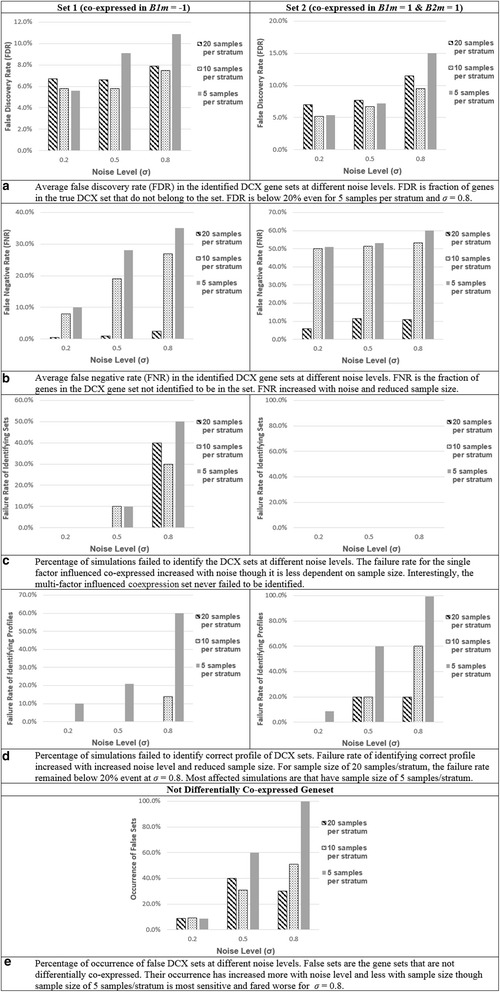
Simulation results. The simulations were carried out for 5 samples/stratum, 10 samples/stratum and 20 samples/stratum. Set 1 represents gene set simulated to be co-expressed only in samples *B1*_*m*_ *= −1*, while Set 2 represents gene set simulated to be co-expressed for *B1*_*m*_ *= 1* and *B2*_*m*_ *= 1* (**a**) FDR, (**b**) FNR, (**c**) Failure rate of identifying DCX genesets, (**d**) Failure rate of identifying DCX profile of DCX genesets, and (**e**) FPR of DCX genesets (non-DCX genesets)

The simulation results are summarized in the panel of plots in Fig. 5: plots of average numbers of false positives (FPs) and false negatives (FNs) over 10 independent simulation runs for each choice of *σ* and sample size. MultiDCoX performed well in terms of both false positives and false negatives for low to medium values of *σ*. Moreover, the algorithm exhibited reasonable performance even at the noise (*σ*) comparable to the signal (i.e. σ = 0.8). The simulation results also demonstrate that MultiDCoX is sensitive even at small sample size for low to medium noise level. The failure rate of identifying genesets and their profiles are dependent not only on the sample size and noise level, but also on the type of set identified, especially for low sample size and high noise: the single factor influenced geneset has better chance of being identified with right factor profile, whereas the set influenced by 2 factors has higher chance of being identified. The effect of noise on FNR also depended on the number of factors influencing the DCX gene set. However, FDR is less dependent on both noise level and the number of factors influencing co-expression. Number of simulations that identified false gene sets increased with increased noise and reduced sample size. It is the lowest for 5 samples/stratum and high noise (*σ* = 0.8). The computational time for MultiDCoX analysis, to optimize each cofactor in both directions (maximization and minimization), was ~12–15 h for one simulated data of 240 samples using 1 node of a typical HPC cluster.

### MultiDCoX analysis of breast tumor data

We analyzed a breast tumor gene expression data published by Miller et al. [23]. It contains expression profiles of tumors from 258 breast cancer patients on U133A and U133B Affymetrix arrays i.e. ~44,000 probes. Tumors were annotated for their oestrogen receptor (ER) status (1 for recognizable level of ER or *ER+*, −1 otherwise or *ER-*), p53 mutational status (1 for mutation or *p53+,* and −1 for wild type or *p53-*) and grade of tumor (−1 for grade 1, 0 for grade 2, and 1 for grade 3). ER and p53 status are important markers used to guide treatment and prognosis of breast cancer patients. Hence it is important to identify the genesets regulated and thereby co-expressed by these factors while accounting for the effect of the tumor status as indicated by its grade and strong association between these three co-factors. For example, p53-mutant tumors are typically of higher grade (grades 2 or 3) tumors with correlation of ~ 63% [24] and ER-positive tumors are typically of low grade (grade 1) [25]. In the presence of these correlations among the co-factors, it is important to identify and quantify their effects on co-expression of gene sets. We applied *MultiDCoX* on this dataset using ER status, p53 mutational status and tumor grade as co-factors. We discuss a few DCX genesets here and the remaining DCX gene sets are given in the Additional file 1.

**Fig. 6:**
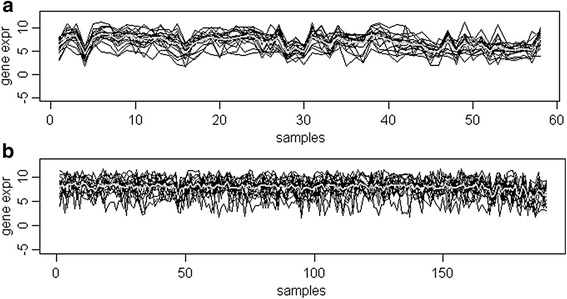
The co-expression plot of set 1 (Table 1) in *p53+* tumors in the breast cancer data. **a** Co-expression of geneset 1 (18 genes) across p53 mutant tumor (p53+) samples; gray color line indicates mean expression value of geneset 1. **b** The geneset 1 showed no co-expression in p53 wild-type samples (p53-); gray color line indicates mean expression value of geneset 1

**Co-expression of ER pathway and the genes associated with relevant processes is modulated in p53 mutated tumors:** A DCX gene set is shown in Table 1. The set is co-expressed only in p53 mutant tumors. The co-expression plot of p53 mutant tumors is shown in Fig. 6.

**Table 1:** A gene set differentially co-expressed by p53-mutational status (*p*-value = *2.75E-231* and coefficient = *1.137*) only and insignificant for the other co-factors: coefficients/*p*-values for ER and Grade are 0.087/0.114 and −0.063/0.028 respectively. Co-expression of the set occurs in p53 mutated tumors only. ER dependent differential expression, ER binding sites and p53 binding sites are also given for the geneset

No.	Gene	ER (DE)	ER Binding Site	p53 Binding Site	Gene Description
1.	GFRA1	Yes (up)	Yes (dist = 58.5 kb)	No	TGF-beta related neurotrophic factor receptor
2.	FOXA1	No	Yes (dist = 4.79 kb)	No	Forkhead box protein A1
3.	GATA3	No	Yes (dist = 30.33 kb)	Yes	GATA binding protein 3
4.	SPDEF	No	Yes (dist = 1.15 kb)	No	SAM pointed domain containing ets transcription factor
5.	ESR1	Yes (up)	Yes (dist = 32.24 kb)	Yes	Estrogen receptor 1
6.	GAMT	No	dist >100 kb	Yes	guanidinoacetate N- methyltransferase
7.	TOX3	No	dist >100 kb	No	TOX high mobility group box family member 3
8.	AGR3	Yes (up)	Yes (dist = 54.06 kb)	No	anterior gradient 3 homolog (*Xenopus laevis*)
9.	SDR16C5	No	dist >100 kb	No	Short-chain dehydrogenase/reductase family 16C member 5
10.	PIP	No	dist >100 kb	No	prolactin-induced protein
11.	CYP2B7P1	No	dist >100 kb	No	cytochrome P450, family 2, subfamily B, polypeptide 7 pseudogene 1
12.	SYTL5	Yes (up)	Yes (dist = 94.21 kb)	No	synaptotagmin-like protein 5
13.	MKX	No	Yes (dist = 35.21 kb)	No	mohawk homeobox
14.	REEP6	No	dist >100 kb	No	receptor accessory protein 6
15.	AGR2	Yes (up)	Yes (dist = 2.15 kb)	No	anterior gradient 2 homolog (Xenopus laevis)
16.	ANKRD30A	No	dist >100 kb	No	ankyrin repeat domain 30A
17.	CA12	Yes (up)	Yes (dist = 56.69 kb)	Yes	Carbonate dehydratase XII
18.	SCGB2A1	No	dist >100 kb	No	secretoglobin, family 2A, member 1

The set includes *ESR1* (which encodes *ERα*), its co-factor *GATA3* and pioneering factor *FOXA1* [26] along with *ER* downstream targets *CA12, SPDEF* and *AGR2*. We retrieved a total of 1349 p53 binding sites’ associated genes data from Botcheva K et al. [27] and Wei CL et al. [28]. p53 binding sites are reported to be close to the promoters of *ESR1* [29] as well as *GATA3*. Furthermore, *GATA3* binds to *FOXA1* [30]. Our finding reinforces the observations made by Rasti et al. [29] that different p53 mutations may have varying effect on the expression of *ESR1* gene, it’s co-factor *GATA3*, pioneering factor *FOXA1* and *SAM-*dependent Mythyltransferase & *p53* interacting *GAMT* which could have resulted in the differential co-expression of the ER pathway. In addition, co-modulation of chromatin structure alternating & *ER* promoter stimulating *TOX3* and Protein transfer associated REEP6 appears to be required to modulate ER pathway by p53.

#### Genes co-expressed with BRCA2 in ER-negative tumors are associated with Her2-neu status:

**Fig. 7:**
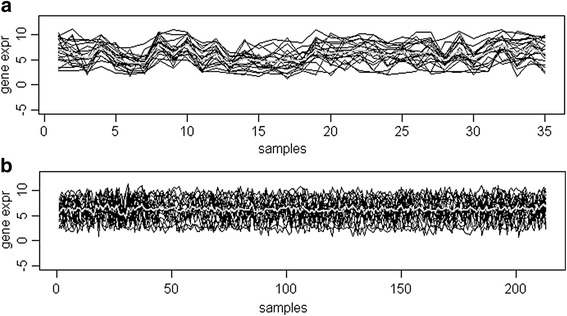
The co-expression plot of set 2 (Table 2) tumors in breast cancer data. **a** Co-expression of geneset 2 (21 genes) in ER-negative tumor samples; gray color line indicates sample-wise mean expression value of the geneset. **b** The geneset 2 showed no co-expression in ER-positive tumor samples; gray line indicates mean expression value

Another gene set of interest is co-expressed in *ER*-negative tumors only and its details are given in Table 2. The co-expression plot of the gene set in *ER*-negative tumors is shown in Fig. 7. The gene set includes tumor suppressor gene *BRCA2*. We have investigated *ER* binding sites published by Carroll et al. [31] and Lin et al. [32] for *ER* binding sites close (within ±35Kb from TSS) to these genes. The ~4800 binding sites mapped to ~1500 genes. Significantly, 10 of the 21 genes in this DCX gene set have *ER* binding sites mapped to them which is statistically significant at F-test *p*-value <0.01. Interestingly, most of these genes have not been identified to be *ER* regulated in the earlier studies using differential expression methodologies, possibly owing to the complexity of regulatory mechanisms. However, many of these genes are down regulated in *ER*-negative tumors. Testing for association of expression of this set with *Her2-neu* status revealed that higher expression in *ER*-negative tumors is associated with *Her2-neu* positivity which must have led to co-expression in *ER*- negative tumors. Odds ratio of such an association is 18 which is much higher than that of *ER* positive tumors (OR = 4).

**Table 2:** A gene set differentially co-expressed by ER-status (*p*-value = 1.34 × 10^−252^ and coefficient = −1.117) only and insignificant for the other cofactors: coefficients/*p*-values for p53 and Grade are 0.294/1.05E-51 and 0.095/9.33E-09 respectively. Co-expression of the set occurs in ER-negative tumors only ER dependent differential expression, ER binding sites and p53 binding sites are also given for the gene set

No.	Gene	ER (DE)	ER Binding Site	Gene Description
1.	BRCA2	Yes (up)	dist >100 kb	breast cancer 2, early onset
2.	ABCC3	Yes (down)	Yes(dist = 20.96 kb)	ATP-binding cassette, sub-family C (CFTR/MRP), member 3
3.	ITGB6	Yes (down)	dist >100 kb	integrin, beta 6
4.	ABCC11	No	Yes(dist = 68.96 kb)	ATP-binding cassette, sub-family C (CFTR/MRP), member 11
5.	SNED1	No	Yes(dist = 94.62 kb)	Insulin-responsive sequence DNA- binding protein 1
6.	NQO1	Yes (down)	Yes(dist = 32.63 kb)	NAD(P)H dehydrogenase, quinone 1
7.	LOC254057	No	NA	uncharacterized LOC254057
8.	SPDEF	No	Yes(dist = 1.159 kb)	SAM pointed domain containing ets transcription factor
9.	FABP4	No	Yes(dist = 1.159 kb)	fatty acid binding protein 4, adipocyte
10.	CEACAM6	*Yes (down)*	Yes(dist = 19.05 kb)	carcinoembryonic antigen-related cell adhesion molecule 6
11.	DUSP4	No	Yes(dist = 19.138 kb)	dual specificity phosphatase 4
12.	SERHL2	No	Yes(dist = 32.63 kb)	serine hydrolase-like 2
13.	RBP4	No	Yes(dist = 20.489 kb)	retinol binding protein 4, plasma
14.	PTK6	*Yes (down)*	dist >100 kb	PTK6 protein tyrosine kinase 6
15.	TMC5	No	dist >100 kb	transmembrane channel-like 5
16.	EEF1A2	No	dist >100 kb	eukaryotic translation elongation factor 1 alpha 2
17.	CLIC3	*Yes (down)*	Yes(dist = 0.317 kb)	chloride intracellular channel 3
18.	LBP	No	dist >100 kb	lipopolysaccharide binding protein
19.	MMP1	No	dist >100 kb	matrix metallopeptidase 1 (interstitial collagenase)
20.	FAM5C	No	dist >100 kb	family with sequence similarity 5, member C
21.	AGR2	Yes (up)	Yes(dist = 2.154 kb)	anterior gradient 2 homolog (Xenopus laevis)

#### DCX of CXCL13 is modulated by grade as well as ER status

Analysis of Grade1 and Grade3 tumors using GGMs [22] helped identify *CXCL13* in breast cancer as hub gene. It emerged as one of the hub genes in our analysis too, contributing to multiple DCX gene sets (see Additional file 1 *,* sheet*:maxGrade*). Although they are significant for Grade, they are significant for ER status too. It shows that CXCL13’s differential co-expression appears to be influenced by ER status, in addition to Grade. This couldn’t be identified in the previous study as it was restricted to single-factor (Grade) analysis.

#### DCX of MMP1 is modulated by factor subspace associated with poor survival

*MMP1* is another gene we have examined whose family of genes are associated with poor survival [33]. MMP1 is co-expressed among tumors which are P53+ (mutant) and ER-negative or hi-grade tumors which are ER-postive (see Additional file 1 *,* sheets*: maxP53, maxGrade and minER*). Both these categories are known to be associated with poor survival of patients. This couldn’t have been revealed in single factor analyses.

#### DCX Modulated by Multiple Factors

Co-expression of many genesets is modulated by more than one factor. The genesets discussed for MMP1 and CXCL13 are examples of such multi-factor DCX i.e. co-expression of these genesets is modulated by ER status and Grade of the tumors. One such set is shown in the 1st row of Table 3. In addition, we presented one geneset whose co-expression is modulated by all factors (covariates): ER status, p53 mutational status and Grade of tumors (ER+ & P53- & Grade+); and, another gene set whose co-expression is modulated by ER status and p53 status (ER- & p53+), Table 3.

**Table 3:** Examples of genesets whose co-expression is influenced by more than one factor. (1) geneset in the 1st row, containing CXCL13 and MMP1, is differentially co-expressed by ER and Grade covariates

Co-expression	Genes	ER coefficient	ER pvalue	p53 coefficient	p53 pvalue	Grade coefficient	Grade pvalue
ER+ & Grade+	HORMAD1,SCGB1D2, ABCB1,IGHM,CXCL13, FAM20B,IGK,CCL18, LOC100291464,FCRL5, IGHA1,LOC100293440, IGL,IGLV1–44,IGH, IGKV4–1, IGHD, LOC100130100,FABP7, NKG7,MMP1,PIGR, LOC652493	0.592	3.64E-05	−0.343	0.000 658	1.525	5.05E-24
ER+ & P53- & Grade+	CLEC3A, MUC5B, RAD51C,CYP2A6,CHGB, CARTPT,GRIA2,INSM1, NTS,PCSK1	0.662	3.16E-05	−0.818	4.14E-17	1.124	1.77E-40
ER- & p53+	FMO5, VGLL1, FABP7, GABRP, PKP1,TFCP2L1, NRTN,KRT15, PTX3, KRT16, MIA,CTAG1A, ELF5,HORMAD1,C8orf4 6,FAM150B	−0.906	7.80E-14	0.844	8.84E-23	0.127	0.305

The co-expression of the set occurs in ER-positive tumors and higher grade tumors (referred to as Grade+) only. Joint occurrence of ER+ and Grade3 will result in higher co-expression. (2) Geneset in the 2nd row is differentially co-expressed by all covariates. The co-expression of the set occurs in ER-positive tumors, p53-negative tumors and higher grade tumors (referred to as Grade+). Joint occurrence of ER+, p53- and Grade3 will result in higher co-expression. (3) Gene set in the 3rd row is differentially co-expressed by ER and p53 covariates. The co-expression of the set occurs in ER-negative tumors, p53-positive tumors. Joint occurrence of ER- and p53+ will result in higher co-expression

#### Functional analysis of DCX profiles

To elucidate the biological function of different DCX profiles (ER+, ER- & p53+, etc.), we pooled all genes from gene sets of same DCX profile and used ClusterProfiler [34], Huang et al. [35] to identify GO terms and pathways enriched. Results for co-expression influenced by individual factors as well as selected two factor combinations are tabulated in Additional file 2. It shows a clear distinction of GO functional categories and pathways enriched between different DCX profiles. For example, many pathways and GO terms are uniquely enriched for single factors. Strikingly, numerous pathways and biological processes/functions are modulated by more than one factor. It couldn’t have been easily deciphered by univariate analyses. Both these observations assert the need for multi-variate analysis of co-expression and such need met by MultiDCoX.

## Conclusions

MultiDCoX is a space and time efficient algorithm which successfully elicits quantitative influence of co-factors on co-expression of gene sets. It required only 12hr of computation on a typical HPC node to identify DCX gene sets for each factor for a dataset of 240 samples and ~44,000 probes. The simulation results demonstrated that MultiDCoX has tolerable false discovery rates even at 5 samples/stratum and noise (*σ*) of 0.8. However, false negative rate (FNR) was affected by both sample size and noise level: FNR is very low for large sample size (20 samples per stratum) and low noise level (*σ* = 0.2). Interestingly, both FDR and FNR did not greatly depended on the type of the gene set to be discovered, or whether it is influenced by single factor or multi-factors. The discovery of a gene set whose DCX is driven by two cofactors is less affected by noise and sample size than the gene sets influenced by a single cofactor. On the other hand, at low sample size and high noise, the set influenced by 2-cofactors has higher likelihood of arriving at the incomplete profile compared to that of a 1-cofactor driven DCX. Occurrence of false DCX sets increased substantially at high noise level and small sample size. This is a major issue to be addressed in the future improvements of MultiDCoX. Moreover, the performance of the algorithm needs to be studied for varying parameter settings and further reductions in computational time. It is possible to reduce the computational time by 2-fold by filtering out 50% of probes of low variance in expression. Though we have not used this strategy as we needed to study its impact on the discovery and profiling of DCX gene sets, the current implementation could complete the analysis within half a day of computing for each factor. The massive parallel processing allows us to complete all analyses within a day.

Though the current implementation of MultiDCoX is limited to linear model, we can easily augment the implementation to use any link function to transform *A(I)* and then using linear function. However, we need to test the performance of the algorithm for various link functions.

By MultiDCoX formulation, we identify DCX gene sets exhibiting B-type co-expression only [22]. The other two types of differential co-expression may be identified using multivariate differential expression analysis followed by clustering.

MultiDCoX algorithm can be applied to different clinical data to quantify the influence of co-factors on the co-expression and its associated phenotypes.

Multiple aspects of the formulation and the algorithm need to be studied in our future improvements: Robustness of *A*_*mn*_ *(I)* to outliers is an important aspect of the performance of the algorithm and impact of the thresholds used in the algorithm also to be studied. However, without tuning, the choice of parameters appears to be effective enough for both simulated and real data sets.

The application of MultiDCoX on a breast cancer data has revealed interesting sets of DCX genes: the set of *ESR1*, its cofactors along with downstream genes of ESR1 and genes associated with relevant ESR1 dependent transcriptional regulation; the set of genes containing ER binding site in their *cis* region. Furthermore, we have shown that the co-expression of gene sets that contain *CXCL13* and the gene sets that contain MMP1 is affected by ER status too, in addition to tumor grade which couldn’t have been elicited in a typical univariate DCX analysis. The utility of MultiDCoX is further demonstrated by revelation of co-expression modulated by multiple factors for numerous genesets and pathways.

## Additional files

Additional file 1: Results of Analysis of Breast Cancer Data. Contains all differentially co-expressed genesets with respective differential co-expression model fit (F-test *p*-value, coefficient value), gene counts, and permutation results over three factors (ER, p53 and Grade) in breast cancer data. Remarks: Grade + indicates higher grade tumor i.e. 2 and 3, while Grade– indicates lower grade tumour i.e. 1. (XLS 804 kb)

Additional file 2: Functional analysis of joint and individual influence of co-factors on co-expression of genesets. Summary of GO terms and pathways enriched for joint and individual influence of different cofactors on co-expression of genests. Joint influence of co-factors is evident from the number of pathways and GO terms enriched for genesets whose co-expression is affected by more than one co-factor. (DOC 66 kb)

## Abbreviations

DCX: Differential Co-expression/Differentially Co-expressed
DE: Differential Expression
ER: Oestrogen Receptor
FDR: False Discovery Rate
FNR: False Negative Rate
FNs: False Negatives
FPR: False Positive Rate
FPs: False Positives
GO: Gene Ontology
HPC: Hi Performance Computing
KEGG: Kyoto Encyclopaedia of Genes and Genomes
OR: Odds Ratio
