# Supplementary material for: MultiDCoX: Multi-factor analysis of differential co-expression
Source: BMC Bioinformatics. 2017 Dec 28;18(Suppl 16):576. doi: 10.1186/s12859-017-1963-7 (PMC5751780; doi:10.1186/s12859-017-1963-7)
Supplement: Supplementary file 2 — Functional analysis of joint and individual influence of co-factors on co-expression of genesets. Summary of GO terms and pathways enriched for joint and individual influence of different cofactors on co-expression of genests. Joint influence of co-factors is evident from the number of pathways and GO terms enriched for genesets whose co-expression is affected by more than one co-factor. (DOC 66 kb) [file 12859_2017_1963_MOESM2_ESM.doc]

## Additional File2 - Functional analysis of co-expression in different covariates

| **Significant Covariates** | **Biological Process** | **Molecular Function** | **Cellular Components** | **Pathway (KEGG)** |
| --- | --- | --- | --- | --- |
| ER+ | anatomical structure morphogenesis, cellular process, organ morphogenesis, cell-cell signaling, regulation of developmental process, response to stimulus, system development, anatomical structure development, regulation of multicellular organismal process, cell differentiation | antigen binding, protein binding, receptor binding, MHC class II receptor activity, ionotropic glutamate receptor activity, extracellular-glutamate-gated ion channel activity, syntaxin binding | extracellular region, membrane-bounded vesicle, cell periphery, neuron projection, axon part, synapse, secretory granule, plasma membrane, extracellular vesicle, extracellular exosome, extracellular organelle | Asthma, long-term potentiation, amphetamine addiction, Rap1 Signaling pathway, Type 1 diabetes mellitus, IBD, intestinal immune network for IgA production |
| ER- | single/multicellular-organism cellular process, developmental process, response to stimulus, anatomical structure development, tissue development, system development, biological regulation, organ development, epithelial cell differentiation, cell proliferation, response to lipid, epithelium development, reproductive process, cellular response to chemical stimulus, response to chemical, reproductive structure development, cell adhesion | protein binding, receptor binding, RNA polymerase II transcription factor activity, sequence-specific DNA binding, receptor binding, glycosaminoglycan binding, heparin binding, sulfur compound binding, regulatory region DNA binding, nucleic acid binding TF activity, MHC class II receptor activity, cell adhesion molecule binding, transporter activity | extracellular region, vesicle, membrane-bounded vesicle, extracellular matrix, extracellular organelle, extracellular exosome, plasma membrane part, integral component of plasma membrane, secretory granule lumen, vesicle lumen | ECM-receptor interaction, Drug metabolism - cytochrome P450, Cell adhesion molecules (CAMs), Protein digestion and absorption, PPAR signaling pathway, Retinol metabolism, Steroid hormone biosynthesis, Type 1 diabetes mellitus, Chemical carcinogenesis, IL-17 signaling pathway, Metabolism of xenobiotic by cytochrome P450, Leishmaniosis |
| P53+ | single/multicellular organism process, cell differentiation, cellular developmental process, gland development, anatomical structure development/morphogenesis, cell-cell signaling, connective tissue development, female sex differentiation, development of primary female sexual characteristics, regulation of developmental process, response to stimulus | MHC class II receptor activity, receptor binding | extracellular space, extracellular region, cellular component, blood microparticle, plasma membrane part | Drug metabolism - cytochrome P450, Amphetamine addiction, Fluid shear stress and atherosclerosis |
| P53- | single-organism cellular process, organ development, organ morphogenesis, tissue development, gland morphogenesis, tube development, gland development, cell-cell signaling, extracellular matrix organization, negative regulation of biological process | None | extracellular region, extracellular space, extracellular matrix, membrane-bounded vesicle, proteinaceous extracellular matrix, vesicle | None |
| Gr+ | single/multicellular organism process, response to stimulus, developmental process, single-organism developmental process, organ development, cellular process, system development, response to external stimulus, anatomical structure development/morphogenesis, cell differentiation, cellular chemical homeostasis, immune response, signaling, tissue development | receptor binding, antigen binding, chemokine activity, calcium ion binding, chemokine receptor binding, MHC class II receptor activity, protein binding, G-protein coupled receptor binding, glycosaminoglycan binding, cytokine activity, heparin binding, RNA polymerase II transcription factor activity, sequence-specific DNA binding | extracellular region, cellular component, vesicle, membrane-bounded vesicle, extracellular organelle, extracellular vesicle extra cellular membrane-bounded organelle, extracellular exosome, plasma membrane region, secretory granule, cytoplasmic vesicle | Pancreatic secretion, Hematopoietic cell lineage, Intestinal immune network for IgA production, Chemokine signaling pathway, Regulation of lipolysis in adipocytes, Type I diabetes mellitus, IL-17  signaling pathway, cytokine-cytokine receptor interaction, prolactin signaling pathway |
| Gr- | Biological process | None | extracellular region, membrane-bounded vesicle, vesicle, extracellular membrane-bounded organelle, extracellular exosome, extracellular organelle, extracellular vesicle | Systemic lupus erythematosus |
| ER- & P53+ | single/multicellular-organism cellular process, developmental process, anatomical structure development, response to stimulus, single/multicellular organismal development, epithelium development, anatomical structure morphogenesis, tissue development, cell differentiation, organ development, response to stimulus, response to lipid, cell proliferation | protein binding, receptor binding, RNA polymerase II transcription factor activity, sequence-specific DNA binding, glycosaminoglycan binding, heparin binding, transcriptional activator activity, RNA polymerase II transcription regulatory region sequence-specific binding, sulfur compound binding, transcription factor activity, RNA polymerase II core promoter proximal region sequence-specific binding, nucleic acid binding transcription factor activity, regulatory region DNA binding | Cellular component, extracellular space/region, membrane-bounded vesicle, vesicle, extracellular matrix, proteinaceous extracellular matrix, extracellular organelle, extracellular vesicle, extracellular exosome, plasma membrane part, integral component of plasma membrane | Drug metabolism - cytochrome P450, cell adhesion molecules (CAMs), PPAR signaling pathway, ECM-receptor interaction, protein digestion and absorption, retinol metabolism, chemical carcinogenesis, metabolism of xenobiotic by cytochrome P450, steroid hormone biosynthesis, IL-17 signaling pathway |
| ER+ & Gr+ | single/multicellular organism process, cellular process, response to stimulus, developmental process, system development, response to external stimulus, organ development, anatomical structure morphogenesis/development, cell differentiation, immune response, organ morphogenesis, immune system process, cell communication, tissue development, cell-cell signaling | receptor/protein binding, calcium ion binding, antigen binding, chemokine activity, chemokine receptor binding, MHC class II receptor activity, glycosaminoglycan binding, ion binding, clathrin binding, lipid binding, cell adhesion molecule binding, heparin binding, cytokine activity, G-protein coupled receptor binding, ionotropic glutamate receptor activity, extracellular-glutamate-gated ion channel activity | extracellular region/space, vesicle, cellular component, membrane-bounded vesicle, extracellular organelle, extracellular vesicle, extracellular exosome, plasma membrane region, cytoplasmic vesicle, neuron part, endomembrane system, neuron projection, secretory granule, membrane-bounded organelle, cell surface, cell projection | Amphetamine addiction, hematopoietic cell lineage, long-term potentiation, intestinal immune network for IgA production, pancreatic secretion, prolactin signaling pathway, asthma, chemokine signaling pathway, metabolism of xenobiotic by cytochrome P450, Regulation of lipolysis in adipocytes, Tyrosine metabolism, Rap1 signaling pathway, IL-17 signaling pathway |
